# Identification and preliminary characterization of *Hc-clec-160*, a novel C-type lectin domain-containing gene of the strongylid nematode *Haemonchus contortus*

**DOI:** 10.1186/s13071-018-3005-3

**Published:** 2018-07-20

**Authors:** Ling Zhang, Lingyun Mou, Xueqiu Chen, Yi Yang, Min Hu, Xiangrui Li, Xun Suo, Xing-Quan Zhu, Aifang Du

**Affiliations:** 1 College of Animal Sciences, Zhejiang Provincial Key Laboratory of Preventive Veterinary Medicine, Zhejiang University, Hangzhou, China; 2 State Key Laboratory of Agricultural Microbiology, College of Veterinary Medicine, Huazhong Agricultural University, Wuhan, China; 3 College of Veterinary Medicine, Nanjing Agricultural University, Nanjing, China; 4 State Key Laboratory of Agrobiotechnology, Key Laboratory of Zoonosis of Ministry of Agriculture, National Animal Protozoa Laboratory and College of Veterinary Medicine, China Agricultural University, Beijing, China; 5 State Key Laboratory of Veterinary Etiological Biology, Key Laboratory of Veterinary Parasitology of Gansu Province, Lanzhou Veterinary Research Institute, Chinese Academy of Agricultural Sciences, Lanzhou, 730046 Gansu Province China

**Keywords:** *Hc-clec-160*, *Haemonchus contortus*, *Caenorhabditis elegans*, C-type lectin

## Abstract

**Background:**

The strongylid parasite *Haemonchus contortus* causes severe anemia in domestic animals worldwide. Effective preventive and therapeutical agents are lacking, because of drug resistance and that little is known about the molecular mechanism of the interaction between *H. contortus* and host cells.

**Methods:**

A new gene, *Hc-clec-160*, was discovered with RT-PCR. Transcriptional levels of *Hc-clec-160* and *Ce-clec-160* throughout different growth phases of corresponding nematodes were assayed by qPCR. Immunofluorescence staining of paraffin section were performed to determine the protein localization in adult worms of *H. contortus*. To monitor the promoter capacity of the 5'-flanking region of *Ce-clec-160*, micro-injection was used. Overexpression and RNAi constructs was carried out in the N2 strain of *Caenorhabditis elegans* to find out the gene function of *Hc-clec-160*.

**Results:**

The full-length cDNA of 1224 bp of *Hc-clec-160* was cloned by RT-PCR. The corresponding gene contained twelve exons. Its transcripts peaked in male adult worms. Hc-CLEC-160 was predicted to have a Willebrand factor type A (vWA) domain and a C-type lectin domain. The proteins were not detected by expression in *C. elegans* or paraffin section experiments in adult of *H. contortus*. Knockdown of *Ce-clec-160* expression in *C. elegans* by RNAi resulted in shortened body length and decreased brood size.

**Conclusions:**

In this experiment, a new gene *Hc-clec-160* was obtained in *H. contortus* and its function was addressed using a model organism: *C. elegans*. Our study showed that *Hc-clec-160* possesses characteristics similar to those of *Ce-clec-160* and plays an important role in the growth and reproduction of this parasitic nematode.

**Electronic supplementary material:**

The online version of this article (10.1186/s13071-018-3005-3) contains supplementary material, which is available to authorized users.

## Background

C-type lectin (CTL) is a Ca^2+^-dependent glycan-binding protein (GBP) that shares primary and secondary structural homology in its carbohydrate-recognition domain (CRD). The CRD of CTL is generally regarded as the CTL domain (CTLD), representing a ligand binding motif that binds to sugars, proteins, lipids and even inorganic ligands [1]. Not all CTLs with this domain bind either glycans or Ca^2+^. The CTLD is defined by amino acid sequences and Cys positions, as well as the folded structure. The latter is characterized by two highly conserved disulfide-stabilized bicyclic structures that participate in binding to sugars with at least four conserved cysteine residues, two to three hydrophobic cores, and up to four calcium binding sites. The main conserved residues that bind to sugars include the EPN (Glu-Pro-Asn) motif which enhances binding to Man, GlcNAc, Fuc, and Glc and the WND (Trp-Asp-Asn) motif which promotes binding to Gal and GalNAc, as seen in mouse L-selectin and rat mannose-binding protein C [1, 2]. They often oligomerize, which increases their avidity for multivalent ligands. CTLs bind to various types of glycans with high affinity. CTLs include selectins, collectins, endocytic receptors and proteoglycans. Some CTLs are exocrine whereas others are transmembrane proteins. They play a role in adhesion and signaling receptors in many pathways, including homeostasis and innate immunity, and are crucial in inflammatory responses and leukocyte and platelet trafficking. It is difficult to predict the glycan structures that bind to a particular CTL due to the relatively shallow CRD of CTL with few contacts to sugars motifs.

CTLs have been identified in some parasitic nematodes such as *Trichostrongylus colubriformis*, *Onchocerca volvulus*, *Haemonchus contortus* and *Teladorsagia circumcincta* [3]. They can significantly regulate host immune response [4–6]; however, their functions in *H. contortus* are poorly understood. *Haemonchus contortus* is one of the most economically important parasites of small ruminants (sheep and goats) worldwide, and can lead to anemia, weight loss and death of the host (sheep and goats) [7]. It can also cause immunosuppression and reduce the level of immunity in the host [8]. *Caenorhabditis elegans* is the most widely used model nematode in drug research [9], vaccine discovery [10] and helminth resistance studies [11–13].

In our study, we identified and characterized a novel gene of *Hc-clec-160* in *H. contortus*, which is an ortholog of *C. elegans* and possesses one CTLD*.* Understanding the structure and function of *Hc-clec-160* may provide useful information for deciphering how this parasite interacts with its hosts and crucial elements influencing its growth, development and reproduction.

## Methods

### Parasites and animals

Two female sheep, 4–5 months-old and maintained under helminth-free conditions for 30 days, were infected orally with 8000 L3s of *H. contortus* ZJ strain each, after being dewormed twice. Three weeks later, sheep feces were collected and helminth eggs detected by microscopy. First- (L1), second- (L2) and third-stage larvae (L3) were harvested at the 1st, 3rd and 7th day, respectively, after incubation of the collected eggs at 28 °C. Adults of *H. contortus* (ZJ strain) were obtained from abomasa of sheep in a Hu Zhou Slaughterhouse. They were washed several times in phosphate-bufferred saline (PBS), pH 7.4. The clean adult worms were stored in liquid nitrogen. Caenorhabditis elegans wild strains (N2) were cultivated on NGM plates and fed on *Escherichia coli* (OP 50 strain) at 20 °C as previously described [14].

### Isolation of the *Hc-clec-160* gene

A homolog (>HCISE02108900.t1) was identified by using the *Ce-clec-160* gene by searching the Sanger Institute genomic database for *H. contortus* (http://www.sanger.ac.uk/cgi-bin/blast/submitblast/h_contortus). Specific primers (*Hc-clec-160* 1F and *Hc-clec-160* 1R) (Table 1) were designed according to the CDs sequence. The PCR reaction program included: one cycle at 94 °C for 3 min; 30 cycles at 94 °C for 30 s, 57 °C for 30 s and 72 °C for 80 s; and finally one cycle at 72 °C for 10 min. The amplified fragments were cloned into the pMD18-T vector (Takara Biotechnology Co., Ltd., Dalian, China) and sent to Shanghai BioSune Co., Ltd. (Shanghai, China) for sequencing.

**Table 1:** Primers used in the study. The restriction sites are underlined

Primer ID	Primer sequence 5'-3'
*Hc-clec-160* 1F	ATGCAACCCCTCTGTACCTT
*Hc-clec-160* 1R	TTAGATGACAGGACAGTAGTGGC
*Hc-clec-160* 2F	GGATCCATGCAACCCCTCTGTACCTT
*Hc-clec-160* 2R	AAGCTTTTAGATGACAGGACAGTAGTGGC
*Hc-clec-160* 3F	TCTAGAATGCAACCCCTCTGTACCTT
*Hc-clec-160* 3R	GGTACCATGACAGGACAGTAGTGGC
*Hc-clec-160* 4F	TCTAGAATGCAACCCCTCTGTACCTT
*Hc-clec-160* 4R	AAGCTTTTAGATGACAGGACAGTAGTGGC
*Ce-clec-160* pF	CCGGACCATCAATATGGTATTGAGTAT
*Ce-clec-160* pR	CTGAATAATTCTTGGGTATTAAAAAAAAGA
*Ce-clec-160* pF1	GTCGACCCGGACCATCAATATGGTATTGAGTAT
*Ce-clec-160* pR1	TCTAGACTGAATAATTCTTGGGTATTAAAAAAAAGA
*Hc-clec-160* QF	AGTAGCGGCTTTGATTGTGC
*Hc-clec-160* QR	TGACTGCTGACCCATTGCTT
*Ce-clec-160* F	CCCGGGATGGATTTAAAAAGTTGGATTCTA
*Ce-clec-160* R	AAGCTTCTAATTGCTTGAAGGTGCACAGTA
*Ce-clec-160* QF	AGTTCCACCTACACATCCG
*Ce-clec-160* QR	AAGTCCACCTTGTCCCATT

### Bioinformatics analyses

Function domains of *Hc-clec-160* gene were analyzed by submitting its amino acid sequence to the NCBI conserved domains (https://www.ncbi.nlm.nih.gov/Structure/cdd/wrpsb.cgi). Homologues of Hc-CLEC-160 in other species: model nematodes (*Caenorhabditis elegans*, *Pristionchus pacificus*), animal parasite (*Ascaris suum*), zoonotic parasites (*Toxocara canis*, *Oesophagostomum dentatum*, *Ancylostoma ceylanicum*) and human parasites (*Necator americanus*, *Ancylostoma duodenale*) were obtained by NCBI Protein BLAST alignment (https://blast.ncbi.nlm.nih.gov/Blast.cgi) and all amino acid sequences were analyzed using Clustal W software [15]. Using evolutionary genetic analysis software MEGA v.7, based on the Jones-Taylor-Thornton (JTT) model, phylogenetic tree analysis was performed using neighbor joining (NJ), maximum parsimony (MP) and maximum likelihood (ML) methods [16].

### Transcriptional levels of *Hc-clec-160* in different developmental stages of *H. contortus*

qPCR with specific primers (*Hc-clec-160* QF-*Hc-clec-160* QR and *Ce-clec-160* QF-*Ce-clec-160* QR) (Table 1) was carried out to determine the mRNA levels in different stages of growth and development of *H. contortus* and *C. elegans* including L1, L2, L3, L4 and adults. Total RNA was isolated with Trizol reagents (Invitrogen, Shanghai, China) according to the manufacturer’s instructions and treated with DNase I (Toyobo, Shanghai, China) to remove the DNA. The qPCR reaction program included: one cycle at 50 °C for 2 min and 95 °C for 1 min; 40 cycles at 95 °C for 15 s, 60 °C for 15 s and 72 °C for 30 s; with a dissolution curve being produced in the last cycle. Each sample was repeated three times using the *β-tubulin* of *H. contortus* and *actin-1* of *C. elegans* as internal reference genes and an average threshold (Ct) was taken for data analysis.

### Polyclonal antibody preparation

Specific primers with double restriction sites (*Hc-clec-160* 2F and *Hc-clec-160* 2R) (Table 1) were designed according to the full-length cDNA of the *Hc-clec-160* gene sequence. *Hc-clec-160* was cloned into the prokaryotic expression vector pET-30a to construct the plasmid pET-30a-*Hc-clec-160*. The vector was transformed into BL21 (DE3) cells of *E. coli* to produce recombinant Hc-CLEC-160 (rHc-CLEC-160), followed by treatment with 1 mM isopropy β-D-1-thiogalactopyranoside (IPTG) at 37 °C for 2 h and purification by affinity chromatography using a Ni-NTA agarose column (Qiagen, Shanghai, China) as per the manufacturer’s instructions. Anti-rHc-CLEC-160 polyclonal antibodies were produced by immunizing a New Zealand white rabbit and their titer and specificity were determined by enzyme-linked immunosorbent assay (ELISA).

### Immunofluorescence staining of *H. contortus* adult paraffin sections

Male and female adults of *H. contortus* were fixed in 4% paraformaldehyde (PFA) at 4 °C for 2 days. After being washed with tap water for 12 h, they were consecutively dehydrated in an ethanol series [50%, 75%, 80%, 95%, 100% (twice), 10 min at each step]. The dehydrated worms were incubated in xylene:absolute ethanol (1:1) solution and xylene and then embedded in paraffin. Paraffin sections of 5 μm were stained with hematoxylin and eosin (H&E) and analyzed histologically. The remaining sections were subjected to immunofluorescence staining. Slices were treated with 0.01 M citrate buffer at 100 °C for 20 min for antigen repair. After overnight blocking with 2% BSA at 4 °C, polyclonal antibody and goat anti-rabbit IgG H&L (Alexa Fluor® 488) diluted 1:100 in PBS were sequentially added and incubated at 37 °C for 1 h each. The sections were stained with 4',6-diamidino-2-phenylindole (DAPI) for 40 min at 37 °C and then observed by fluorescence microscopy.

### *Ce-clec-160* promoter transformation of *C. elegans*

The *Ce-clec-160* promoter was amplified by PCR using primers *Ce-clec-160* pF1 and *Ce-clec-160* pR1 (Table 1) containing restriction sites *Sal*I or *Xba*I. The primers were designed according to the 2000 bp upstream of the start codon of *Ce-clec-160*. The PCR products were cloned into the multiple cloning sites upstream of the *gfp* gene of pPD95.77 eukaryotic expression vector, resulting in the plasmid pPD95.77*-Ce-clec-160*-prom. The latter was microinjected into the gonads of *C. elegans* along with another plasmid pRF4 carrying the *rol-6* gene at a final concentration of 50 μg/ml each. The GFP expression of F2 progeny with the phenotype of roller was observed by fluorescence microscopy.

### Expression of Hc-clec-160 in *C. elegans*

Microinjection was performed as previously described [17]. *Hc-clec-160* was amplified based on the specific PCR primers *Hc-clec-160* 3F and *Hc-clec-160* 3R listed in Table 1 with *Xba*I/*Kpn*I restriction enzyme sites. The amplified fragments were cloned into the plasmid pPD95.77 vector between the *Ce-clec-160* promoter region and the *gfp* region. The recombinant plasmid CeP-pPD95.77-*Hc-clec-160* with CeP-pPD95.77-*Ce-clec-160* as a control was microinjected into N2 strains as described above. The F2 progeny with the roller phenotype were collected for further analysis.

### *Hc-clec-160* RNA interference

*Ce-clec-160* and *Hc-clec-160* genes were amplified by PCR with specific primers *Hc-clec-160* 4F-*Hc-clec-160* 4R and *Ce-clec-160* F-*Ce-clec-160* R, respectively (Table 1). The primer pairs contained the restriction sites for *Xba*I/*Hind*III and *Sma*I/*Hind*III, respectively. The amplified fragments were cloned into the L4440 vector, resulting in the recombinant plasmids L4440-*Ce-clec-160* and L4440-*Hc-clec-160*. The latter were transformed into *E. coli* HT115 (DE3) strain with the empty L4440 vector as a negative control. A feeding RNAi experiment was performed according to the classic bacterial feeding manuals [18] with three repeats. The transcriptional levels of RNAi worms were detected by qPCR and the RNAi worms were selected for further phenotype analysis.

### Statistical analyses

Statistical analysis for *Hc-clec-160* and *Ce-clec-160* transcriptional levels and parameters were carried out in Excel (v.10.1.0.7022). Graphs were performed using the GraphPad Prism5.0. *P*-values ≤ 0.05 were considered statistically significant.

## Results

### Characterization of cDNA and phylogenetic analysis of amino acid sequences

**Fig. 1:**
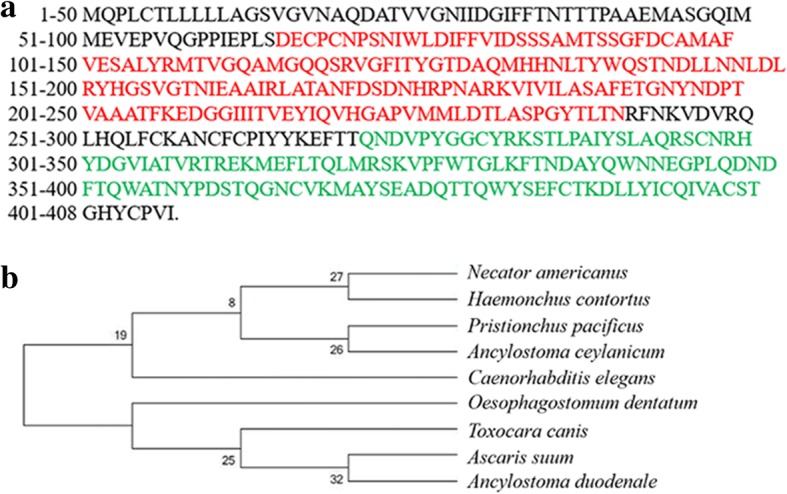
Characterization of *Hc-clec-160* gene from *H. contortus*. **a** Amino acid sequence of *Hc-clec-160*. The conserved domains of Hc-CLEC-160 are highlighted in different colors: the Von Willebrand factor type A (vWA) domain is highlighted in red and the C-type lectin carbohydrate-recognition domain (CRD) in green. **b** A neighbor-joining phylogenetic tree showing the relationship of *H. contortus* C-type lectin domain and its homologues from other nematode species: AAY58318.1 (*Necator americanus*), KKA67531.1 (*Pristionchus pacificus*), EPB71235.1 (*Ancylostoma ceylanicum*), NP_492949.1 (*Caenorhabditis elegans*), KHJ87598.1 ( *Oesophagostomum dentatum*), KHN72314.1 (*Toxocara canis*), ERG83551.1 (*Ascaris suum*) KIH63097.1 (*Ancylostoma duodenale*). The tree was constructed using the Jones-Tayloe-Thornton model in the program MEGA v.7.0

Identification and sequence analysis of the *Hc-clec-160* gene revealed 1224 bp that encoded a 408-amino-acids protein with a predicted mass of 45.2 kDa. *Hc-clec-160* of *H. contortus* contained 12 exons separated by 11 introns, which was structurally similar to *Ce-clec-160* of *C. elegans*. Structural analysis revealed that Hc-CLEC-160 has 2 conserved domains: the von Willebrand factor type A (vWA) domain and the CTL conserved domain, carbohydrate-recognition domain (CRD) (Fig. 1a). The protein sequences of *Hc-clec-160* and another 8 nematodes were phylogenetically analyzed. The phylogenetic tree revealed that Hc-CLEC-160 clustered within the clade containing *C. elegans*, which suggest that their evolutionary relationship is relatively close (Fig. 1b).

### Transcriptional level of *clec-160* throughout the life-cycles of *H. contortus* and *C. elegans* and its protein localization

**Fig. 2:**
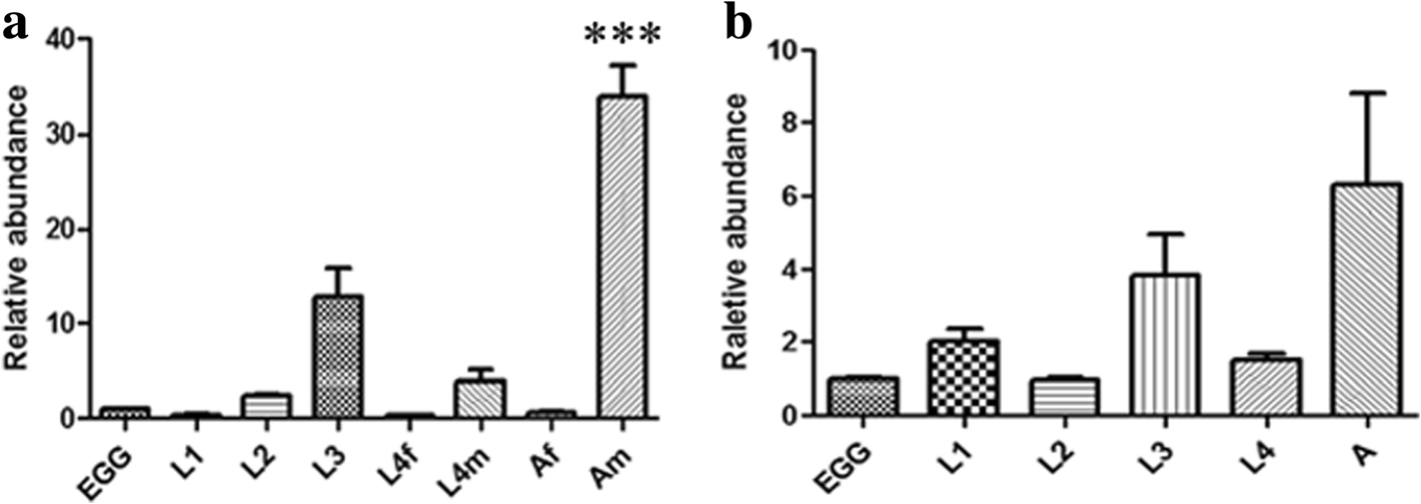
Transcriptional levels of *Hc-clec-160* (**a**) and *Ce-clec-160* (**b**) throughout different developmental stages of *H. contortus* and *C. elegans*. The abundance of *clec-160* transcripts was quantified by qPCR in different developmental stages or sexes of *H. contortus* and *C. elegans*: first-stage larvae (L1), second-stage larvae (L2), third-stage larvae (L3), female fourth-stage (L4f), male fourth-stage (L4m), adult female (Af) and adult male (Am). ***P < 0.001

The transcriptional level of *clec-160* was performed throughout the life-cycles of *H. contortus* and *C. elegans* by qPCR. The result showed that adults had highest levels followed by L3 (Fig. 2). In addition, the transcriptional levels between male and female adults were particularly different (t-test: *t*_(16)_ = 19.81, *P* < 0.0001) with adult males showing much higher transcriptional levels than adult females (Fig. 2a). The results of H&E staining of the sections showed that, regardless of the sex of adults *H. contortus*, parasite morphology was well maintained after sectioning and the tissue boundaries were clear (Additional file 1: Figure S1). Immunofluorescence staining of paraffin sections to detect protein localization was carried out, but no fluorescence staining was observed.

### Expression of Hc-clec-160 in *C. elegans*

**Fig. 3:**
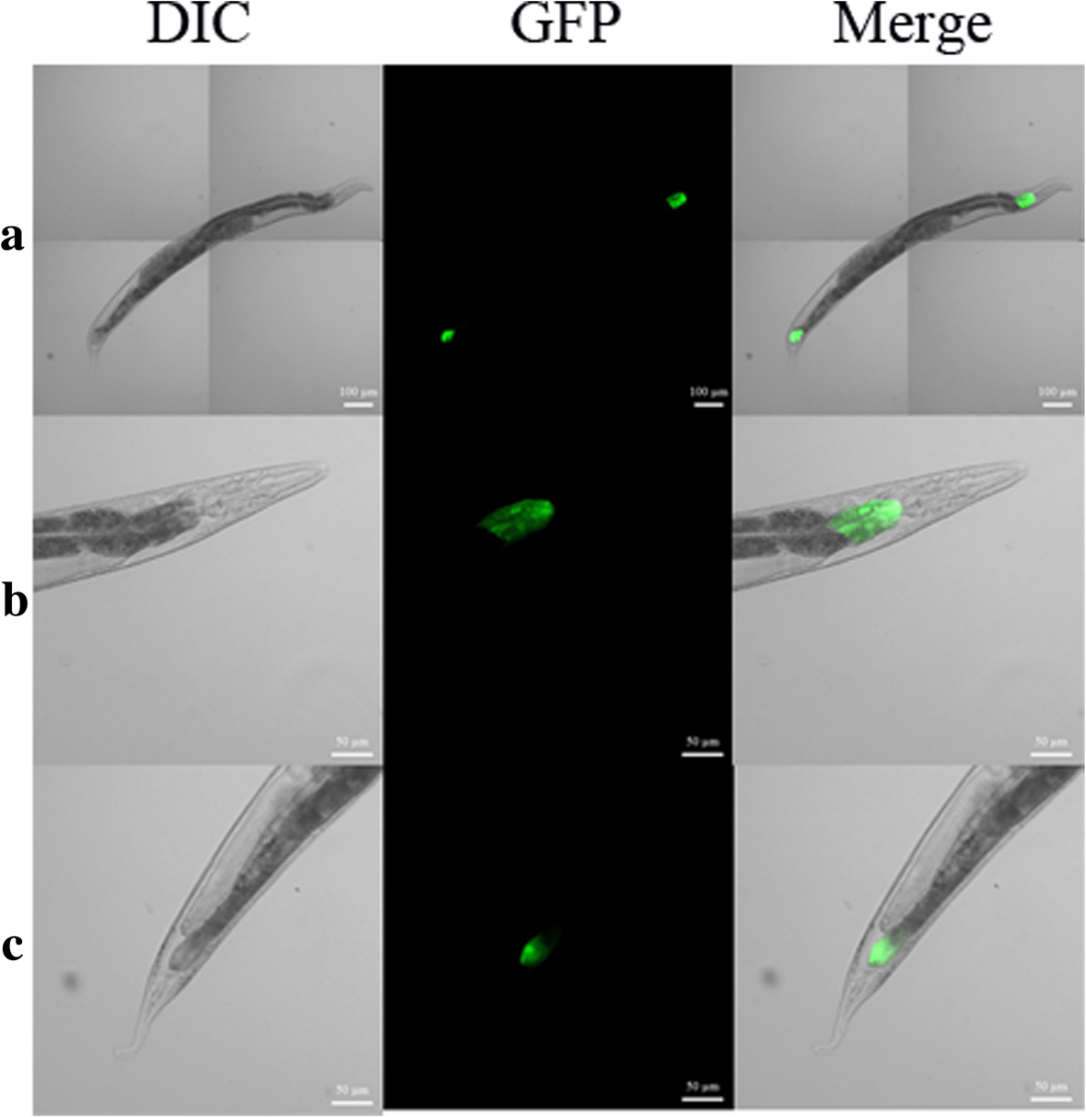
Expression patterns of *Ce-clec-160* promoter in *C. elegans*. **a** Transgene worm at a magnification of 20×. **b**, **c** Anterior and posterior extremities at a magnification of 40×. *Scale-bars*: **a**, 100 μm; **b**, **c**, 50 μm

The entire coding regions of *Hc-clec-160* fused in frame with the *gfp* gene was expressed in transgenic *C. elegans* to detect its expression *in vivo*. *Ce-clec-160* 5'-flanking region was used as a promoter. Activity analysis of the promoter showed that GFP was mainly localized in the distal and anterior part of the intestine (Fig. 3). The reconstructed plasmid Cep-PD95.77-*Hc-clec-160* and Cep-pPD95.77-*Ce-clec-160* was transformed into the gonads of *C. elegans* by microinjecting as described above. However, neither *Hc-clec-160* nor *Ce-clec-160* expression worms showed GFP signal in any stage of *C. elegans*.

### RNAi in *C. elegans*

**Fig. 4:**
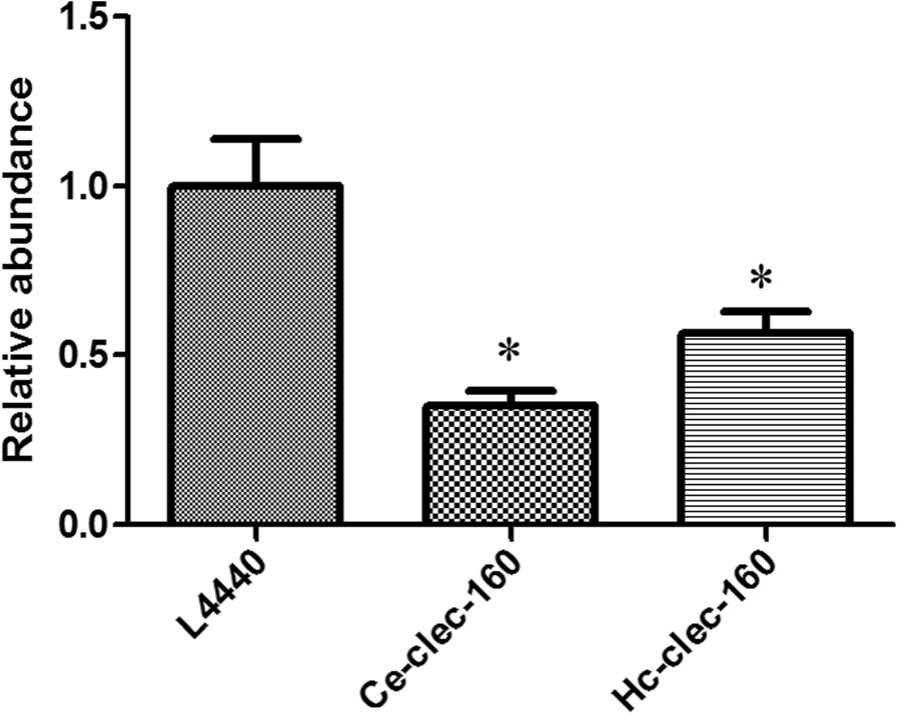
Change in mRNA levels of RNA interference with L4440-*Hc-clec-160* and L4440-*Ce-clec-160* in N2 *C. elegans*. The abundance of *Ce-clec-160* transcripts in *Ce-clec-160* RNAi worms was quantified by qPCR. *P < 0.05

**Fig. 5:**
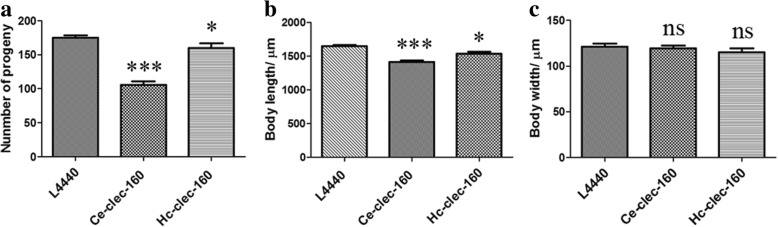
Post-embryonic development in *Ce-clec-160* knockdown *C. elegans* worms. **a** Brood size of twenty worms for three repeats of *Ce-clec-160* RNAi worms, using L4440 “empty” vector as a negative control. **b** Body length of twenty worms for three repeats of *Ce-clec-160* RNAi worms. **c** Body width of twenty worms for three repeats of *Ce-clec-160* RNAi worms. *P < 0.05, ***P < 0.001. *Abbreviation*: ns, difference not significant

Transcriptional levels of the RNAi worms were detected using qPCR to testify whether *Ce-clec-160* in *C. elegans* was successfully interfered. It was confirmed that the transcriptional levels of *Ce-clec-160* of the worms fed with the *Hc-clec-160* and *Ce-clec-160* interference vector were significantly decreased in *C. elegans* by 45% (t-test: *t*_(4)_ = 2.846, *P* = 0.0466) and 65% (t-test: *t*_(4)_ = 4.436, *P* = 0.0114), respectively (Fig. 4). The RNAi worms revealed significant reduction in brood size (t-test: *t*_(19)_ = 2.428, *P* = 0.0253; t-test: *t*_(19)_ = 9.878, *P* < 0.0001) (Fig. 5a) and shortened body length (t-test: *t*_(19)_ = 2.765, *P* = 0.0123; t-test: *t*_(19 )_= 5.986, *P* < 0.0001) (Fig. 5b) compared with the control group; however, the body width of *Ce-clec-160* RNAi worms showed no significant differences from the control (Fig. 5c).

## Discussion

In this study, the full-length cDNA sequences of two genes (*Hc-clec-160* and *Ce-clec-160*) were determined. CTLs are highly representative of all studied metazoan phyla to date. Many proteins in the CTL superfamily contain multiple CRDs, along with additional non-lectin domains. CTLDs were classified to seventeen groups; *Hc-clec-160* was similar to the group XIII DGCR2 (DGCR2/ DD/ Sez 12), which is localized in the DiGeorge syndrome (OMIM 188400) critical region [1]. In von Willebrand factor, the vWF is the archetype for a protein superfamily. The structure of vWF was discovered in various plasma proteins: complement factors B, C2, CR3 and CR4; the integrins (I-domains); collagen types VI, VII, XII and XIV; and other extracellular proteins [19, 20]. Most of the proteins containing vWF domains are exocrine. The vWF-containing proteins are involved in many biological functions such as cell adhesion, pattern formation, migration, homing, and signal transduction, concerning interactions with a large number of ligands. It is plausible that the interaction between lectins of gastrointestinal nematodes and complex mucin oligosaccharides can affect worm infection [21]. It has been reported recently that a CTL named *cpclec* from *Cryptosporidum parvum* plays an important role in binding sulfated proteoglycans of host cells [22, 23]. These findings suggest that Hc-CLEC-160 may have the ability to adhere and bind to host cells.

The relative abundance of *Hc-clec-160* transcripts throughout different developmental stages determined by qPCR showed that they had generally low abundance in all life-cycle stages except for adult males (Fig. 2a). High transcriptional levels of *Hc-clec-160* in the male adult stage suggests that *Hc-clec-160* may be related to the mating and reproduction of *H. contortus*. Furthermore, higher *Hc-clec-160* transcriptional levels in L3s and L4s than in eggs, L1s and L2s, indicate that *Hc-clec-160* is likely to play a crucial part in the parasitic stages, i.e. L3s and L4s, for adhesion to the host cells. The microinjection of *Ce-clec-160* promoter results indicated that this promoter was capable of promoting the GFP expression in *C. elegans* with GFP proteins being mostly located in the anterior and distal part of the intestine.

As for paraffin section immunofluorescence staining and microinjection transgene assays, the possible causes for the lack of fluorescence in *H. contortus* or GFP in *C. elegans* could be summarized as follows. First, the polyclonal antibodies can be detected by western blot rather than the immunofluorescence staining of sections, which may be because the structure of the parasite antigen immobilized by paraformaldehyde is different from the linear structure in the western blot experiment. Secondly, it is possible that the antigen of CTL was damaged during the production of the slices, resulting in the fact that the antibody cannot specifically recognize and bind to it. Thirdly, the GFP expressed in the worms microinjected with *Ce-lec-160* promoter, indicating that the promoter was able to efficiently initiate the expression of GFP. However, the ability to drive the expression of *clec-160* gene was weak. The most likely reason for the failures of these two experiments is due to the rather low expression levels of *clec-160* in *H. contortus* and *C. elegans*.

The mechanism of RNAi is divided into two procedures. First, dsRNA (double stranded RNA) is processed into small ds RNA (siRNA) of 21 to 23 nt. Then, the antisense strands of siRNA induce a ribosome called RNA-induced silencing complex (RISC), which can target homologous mRNA to silence the gene. *Hc-clec-160* and *Ce-clec-160* were ligated into the L4440 plasmid vector with a bidirectional T7 promoter at both ends of the inserted gene. L4440-*Hc-clec-160* and L4440-*Ce-clec-160* were transformed into *E. coli* HT115 (DE3) to induce dsRNA with IPTG. Later on, large numbers of RNAi knockdown worms were produced by feeding *C. elegans* on dsRNA-expressing bacteria. The abundance of *Ce-clec-160* transcripts as a result of RNA interference was determined by qPCR. The relative abundance of mRNA indicates that *Hc-clec-160* can partially silence *Ce-clec-160*, further confirming the genetic structure and possible functional similarities between the two homologous genes. Furthermore, the *Ce-clec-160* (RNAi) phenotype showed much less abundant progeny and shorter body length than negative control worms, which indicates that Ce-CLEC-160 plays a significant role in the growth and development, and reproduction of *C. elegans.*

Helminth parasites possess the ability of modulating the host defense system in order to establish long-term infections in mammalian hosts [24, 25]. The glycans of schistosome eggs can be recognized by L-SIGN [26], DC-SIGN [27] and other members of the CTL family of its hosts, suggesting that the CLRs constitute an ancient host innate immune pattern recognition system. The parasite itself also has CTLs [28, 29], raising the possibility that they may affect host immunity [25, 30]. CTLs can also perform other functions within the nematode phylum. Most notably, *C. elegans* is predicted to have a minimum of 278 CTL-like genes in its genome and some have been experimentally certified to be upregulated and protective in bacterial infections. One example is CLEC-50 [31].

Hc-CLEC-160 is a new protein and, to our knowledge, its function has not been studied so far. In this study, we cloned the *Hc-clec-160* gene and first analyzed its structures. The possible roles of *Hc-clec-160* in *H. contortus* attachment to and invasion of host cells, and also in the escape of host immunity, remain to be determined experimentally. It is very challenging, if not impossible, to directly study *H. contortus* growth and development and reproduction *in vitro* due to the necessity of culturing L3s, L4s and adults. Nevertheless, the predicted structural features and characteristic of *Hc-clec-160* gene reported in the present study along with the known functions of CTL- and CTLD-containing proteins in other organisms including the model nematode *C. elegans* implicate that *Hc-clec-160* may play a significant role in *H. contortus* invasion and the host-parasite cell interactions.

## Conclusions

In the present study, a novel gene *Hc-clec-160* encoding a cDNA of 1224 bp in *H. contortus* was identified. The gene possesses one conserved domain of carbohydrate-recognition domains (CRDs) and contains 12 exons. A possible promoter in the 2000 bp sequence upstream of the 5'-flanking region was demonstrated by microinjection experiments. Furthermore, partially silencing the *Ce-clec-160* in N2 worms of *C. elegans* achieved by RNAi with *Hc-clec-160* showed that *Hc-clec-160* shared similar characteristics and functions with *Ce-clec-160* and plays a crucial part in the development and reproduction of *H. contortus*.

## Additional file

Additional file 1: **Figure S1.** Morphology and histology of male (**a**) and female (**b**) adults of *H. contortus*. Panels **a** and **b** represent H&E staining of worm paraffin sections at 10×. *Scale-bars*: 10 mm. (TIF 1691 kb)

## Abbreviations

BSA: Bovine serum albumin
cDNA: Complementary deoxyribonucleic acid
CRD: Carbohydrate-recognition domain;
CTL: C-type lectin
CTLD: C-type lectin domain
DAPI: 4',6'-diamidino-2-phenylindole
dsRNA: Double stranded RNA
ELISA: Enzyme-linked immunosorbent assay
EPN: Glu-Pro-Asn
Fuc: Fucose
Gal: Galactose
GalNAc: N-acetylgalactosamine
GBP: Glycan-binding protein
GFP: Green fluorescent protein
Glc: Glucose
GlcNAc: N-acetyl-D-glucosamine
H&E: Haematoxylin and eosin
IPTG: Isopropy β-D-1-thiogalactopyranoside
JTT: Jones-Taylor-Thornton
Man: Mannose
ML: Maximum likelihood
MP: Maximum parsimony
mRNA: Messenger RNA
N2: Wild type
NGM: Nematode growth media
NJ: Neighbor-joining
PBS: Phosphate-buffered saline
PCR: Polymerase chain reaction
PFA: Paraformaldehyde
qPCR: Real-time quantitative PCR
rHc-CLEC-160: Recombinant Hc-CLEC-160
RNA: Ribonucleic acid
RNAi: RNA interference
RT-PCR: Reverse transcription PCR
siRNA: Small interfering RNA
vWA: Von Willebrand factor type A
vWF: Von Willebrand factor
WND: Trp-Asp-Asn
