# Supplementary figures and images for: Identification and preliminary characterization of Hc-clec-160, a novel C-type lectin domain-containing gene of the strongylid nematode Haemonchus contortus
Source: Parasit Vectors. 2018 Jul 20;11:430. doi: 10.1186/s13071-018-3005-3 (PMC6054721; doi:10.1186/s13071-018-3005-3)

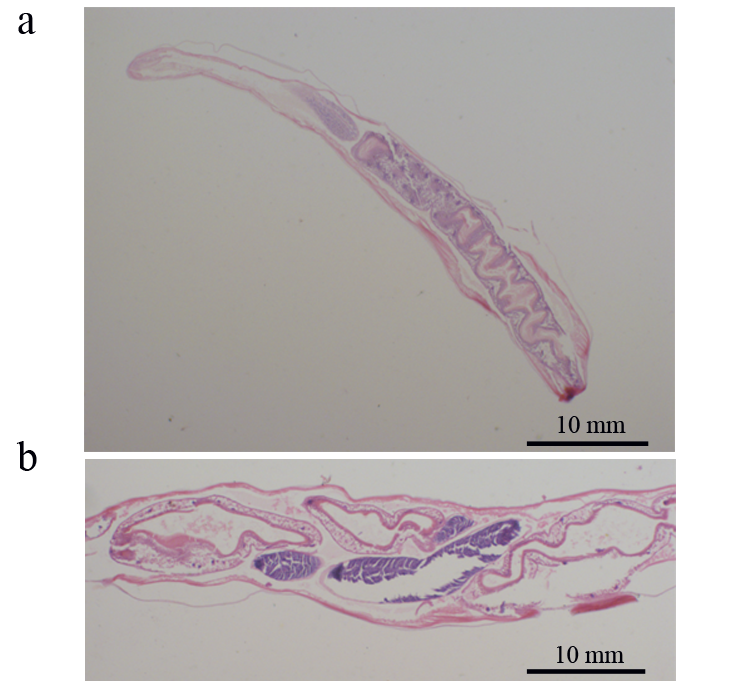

Supplement: Supplementary file 1 — Figure S1. Morphology and histology of male (a) and female (b) adults of H. contortus. Panels a and b represent H&E staining of worm paraffin sections at 10×. Scale-bars: 10 mm. (TIF 1691 kb) [file 13071_2018_3005_MOESM1_ESM.tif]
